# Evaluation of *in vitro* and *in vivo* anti-inflammatory activity of biologically active phospholipids with anti-neoplastic potential in porcine model

**DOI:** 10.1186/1472-6882-14-339

**Published:** 2014-09-19

**Authors:** Monika Vicenova, Katerina Nechvatalova, Katarina Chlebova, Zdenka Kucerova, Lenka Leva, Hana Stepanova, Martin Faldyna

**Affiliations:** Department of Immunology, Veterinary Research Institute, Hudcova 70, 62100 Brno, Czech Republic

**Keywords:** Inflammation, Lipopolysaccharide, Porcine model, Macrophages, Ether phospholipids

## Abstract

**Background:**

This study aims to investigate the anti-inflammatory effect of biologically active phospholipids (BAP) used in preparations for clinical practice in humans. Until date, except anti-neoplastic ability, little is known about anti-inflammatory property of the phospholipids.

**Methods:**

While the course of bacterially induced acute pneumonia and markers of inflammation were studied in *in vivo* system in pigs orally supplemented with BAP, the pro- and anti-inflammatory response of lipopolysaccharide-stimulated porcine monocyte-derived macrophages to 24 h- and 48 h-treatmeant by BAP was investigated in *in vitro* system. *In vivo*, the animal health status was monitored and pro-inflammatory IL-1β and IL-8 in sera were detected by ELISA during the experiment, while bronchoalveolar lavage fluids (BALF) and the lungs were examined post-mortem. Total and differential counts of white blood cell (WBC) were determined in blood and BALF. *In vitro*, mRNA expression of pro-inflammatory (TNF-α, IL-1β, CXCL10) and anti-inflammatory (IL-10 and Arg1) cytokines, and level of activated caspase 1 and phosphorylated protein kinase C epsilon (pPKCϵ), were studied using qRT-PCR and Western blot, respectively. For the purposes of both systems, 6 animals were used in each of the BAP-supplemented and the control groups.

**Results:**

*In vivo*, BAP had a positive influence on the course of the disease. The immunomodulatory effects of BAP were confirmed by lower levels of IL-1β, IL-8, and a lower WBC count in the supplemented group in comparison with the control group. A lower percentage of lung parenchyma was affected in the supplemented group comparing to the control group (on average, 4% and 34% of tissue, respectively). *In vitro*, BAP suppressed mRNA expression of mRNA for IL-10 and all pro-inflammatory cytokines tested. This down-regulation was dose- and time-dependent. Arg1 mRNA expression remained unaffected. Further dose- and time-dependent suppression of the activated caspase 1 and pPKCϵ was detected in macrophages when treated with BAP.

**Conclusions:**

Our results demonstrate that BAP has anti-inflammatory and immunomodulatory properties, thus emphasizing the potential of this compound as a natural healing agent.

## Background

Ether phospholipids (EP) are important constituents of eukaryotic cell membranes and energy reservoir, participating in cell signalling events [1]. The activity of EP leading to the selective destruction of neoplastic cell membranes has been shown in numerous publications over the last decades [2–10] and thus represents a promising tool for cancer therapy for humans. A membrane-tropic mechanism of EP was reported to play a key role in the destruction of neoplastic cell membranes resulting in cell death [11] and the importance of plasma membrane fluidity modulation by lipid membrane composition was presented [12–14]. Moreover, the cytotoxic effect of EP could partially be due to the inhibition of protein kinase C (PKC) or other membrane–associated enzymes [9, 15, 16], which play central roles in cellular signal processing and are involved in the regulation of cell proliferation and migration. PKC was also demonstrated to be an important signalling molecule in cancer invasiveness and metastasis [17, 18]. Considering HeLa cells under physiological conditions, only PKC isoform ϵ was shown to be responsible for their spreading [19]. However, a disadvantage of EP application for human cancer therapy is that most of them are synthetic preparations associated with toxic side effects (such as a membrane destruction of normal, i.e. non neoplastic, cells) limiting their clinical use [3, 7].

A natural mixture of EP prepared from ischemic chick embryonic tissue and described by Kára and coworkers [20] was proved to suppress proliferation and growth of malignant cells with sparing normal physiological cells. This discovery has recently resulted in registration of a unique mixture of biologically active phospholipids (BAF®) (Areko, Inc., Prague, CZ), which is the substantial active component of a commercial pharmaceutical dietary supplement. In accordance with the secret manufacturing process, the exact composition of the mixture cannot be provided. However, it can be affirmed that the natural EP 1-*O*-octadecyl-2oleoyl-*sn*-glycero-3phospho-(*N*-palmitoyl) ethanolamine, i.e., plasmanyl-(N-acyl)-ethanolamine (PNAE) [21], represents 30% of the mixture. A PNAE analogue was semisynthetically prepared [22] and its inhibiting effect on PKC which is of high importance in cancer cell proliferation was established [15]. Both EP, natural and synthetic, have been widely investigated for their selective antineoplastic activity without a toxic effect on normal cells in biological systems *in vitro* and *in vivo* [7, 14, 23, 24]. Another essential component of BAF® is egg phosphatidylcholine (PC) (60%) – one of the major phospholipids in lecithin. PC is known to be the most abundant phospholipid component in eukaryotic cells, being spontaneously organized into bilayers in the outer leaflet of the cell membrane [1].

The aim of this study was to assess the expected anti-inflammatory activity of BAP on the course of experimental infection with *Actinobacillus pleuropneumoniae* (App) in pigs. The activity of BAP was also assessed *in vitro* by evaluating the intensity of inflammatory reaction at the cellular level in a dose- and time- dependent manner.

## Methods

### *In vivo*experiment

#### Animals

A total of 12 piglets included in the study (5 weeks old, average body weight 10–12 kg) were allocated into two groups of 6 animals each. The piglets originated from a herd which was free of clinical App infection for at least 12 months. Neither the piglets nor their mothers were vaccinated against App and the results of anti-App antibody detection in piglet sera by ELISA method [25] were negative.

### Supplementation regime and experimental *Actinobacillus pleuropneumoniae*infection

Animals in experimental group were given a 15% solution of BAP in sunflower oil in a dietary supplement Ovosan (Areko, Inc., Prague, Czech Republic, approximately 75 mg/kg of b.w.). Animals in control group were given pure sunflower oil orally using a syringe, in a volume of 5 ml per animal twice daily for a period of 28 days, with the same time and dosage regime. Fourteen days after the start of the administration of BAP or pure oil, all animals were experimentally infected with 3 ml of the bacterium App via the intranasal route (1.5 ml was administered into each nostril). The field strain KL2–2000, biotype 1, serotype 9 was used at the final concentration of the infectious dose of 4.4 × 10^8^ CFU/ml [26].

### Phospholipids

Phospholipids (PLs) constituting the BAP preparation were extracted from hen egg yolk with ethanol and purified using acetone precipitation according to standard procedures [27]. BAP preparation was enriched to final concentration 30% with 1-*O*-octadecyl-2oleoyl-*sn*-glycero-3phospho-(*N*-palmitoyl) ethanolamine as described earlier [21, 28]. BAP preparation was provided by company Areko, Inc., Prague, Czech Republic.

### Parameters of animal health status investigated during the experiment

The health status of animals was monitored during the entire experiment. Body temperature was taken in the rectum of animals at four clinically important time points: one day before experimental exposure to the infectious agent and at 1, 3 and 7 days post-infection (PI). After App infection, clinical signs of the disease (increased respiration rate, dyspnea, cough, anorexia, lethargy, death) were monitored and recorded twice a day.

Furthermore, peripheral blood samples were collected for determination of total and differential counts of WBC and serological analyses [25] at six time points - before the beginning of supplementation, immediately before infection, and at days 1 (without App antibody detection), 3, 7 and 14 post-infection (PI). Besides that, at first four of these junctures, peripheral blood samples were collected for the detection of acute phase cytokines (interleukins 1β and 8) using a commercially available ELISA kit (Alpco Diagnostic, Salem, NH) in accordance to manufacturer’s recommendation. After completing the experiment (at 2 weeks PI), animals were weighed, euthanized with the intravenous application of anaesthetic T61 (Intervet International B.V., Boxmeer, the Netherland) on the basis of the actual body weight of an animal according to the manufacturer’s recommendations (5 ml/50 kg of body weight), and necropsied. The status of the lung parenchyma was assessed at necropsy and pulmonary scores documenting the extent of pulmonary parenchymal damage were calculated [26]. At the same time, bronchoalveolar lavage fluids (BALF) were collected for serological analysis and cytology of cell infiltrate [29].

### Animal welfare treatment

Animal experiments complied with Act No. 246/92 Sb. and were approved by the Branch Commission for Animal Welfare of the Ministry of Agriculture of the Czech Republic (No. 25–2009; Reg. No. 1092). Experiments were conducted in accredited special barrier facilities for animal housing at the Veterinary Research Institute (Accreditation No. 5843/2007-10001). Animal cadavers were disposed of in compliance with the Rules for Working in experimental animal facilities and valid waste regulations.

### *In vitro*experiment

#### Monocyte-derived macrophages preparation

A mononuclear fraction of white blood cells (WBC) was isolated from the whole heparinized blood from 6 healthy adult pigs using a density gradient technique (Histopaque 1.077, Sigma-Aldrich, St. Louis, MO). Subsequently, a CD14-positive cell subset was selected by indirect magnetic labeling on QuadroMACS™ cell separator (Miltenyi Biotec, Gladbach, Germany) using monoclonal antibody against CD14 (clone MIL2, AbD Serotec, Oxford, UK, 10 μl per 10^8^ cells). CD14-positive cells were captured by goat anti-mouse IgG MicroBeads (Miltenyi Biotec, Gladbach, Germany). The cell subset purity was assessed using flow cytometer LSRFortessaTM (BD Biosciences, San Jose, CA) and was more than 95% in all cases. CD14-positive monocytes, approximately 0.5 × 10^6^ cells per well in 24-well plates, were cultured in Dulbecco’s Modified Eagle’s Medium (Invitrogen, Paisley, UK) supplemented with antibiotics (100,000 IU/l penicillin; 10 mg/l streptomycin; 4 mg/l gentamicin) and 10% (v/v) heat-inactivated porcine serum (PAA Laboratories, Pashing, Austria) at 37°C in an atmosphere with 5% (v/v) CO_2_. After 6 days of cultivation, monocyte-derived macrophages (MDMF) were prepared [30].

### Cell viability determination

The activity of a cytoplasmic enzyme lactate dehydrogenase (LDH), actually its outflow into extracellular space, was monitored. LDH accumulated in culture medium, indicative of increased plasma membrane damage, correlating with the increase in the number of lysed cells, was measured using CytoTox 96® Non-Radioactive Cytotoxicity Assay (Promega, Madison, WI) following the manufacturer’s recommendations.

### Design of culture experiment

The experimental conditions were arranged as follows: cultures of MDMF were incubated with BAP at concentrations 0% (control), 0.03%, 0.1%, and 0.3% (i.e. 0.3; 1; 3 mg per ml of culture media, respectively) for 24 and 48 h. After one following washing step with the cultivation medium, one half of the cultures exposed to the indicated concentrations of BAP was stimulated with 1 μg/ml of lipopolysaccharide (LPS) for 4 hours. Afterwards, the supernatants were removed by aspiration and the adhered cells were lysed in TRI Reagent RT (Molecular Research Center, Inc., Cincinnati, OH) or in 1x Laemmli buffer (0.5 M Tris–HCl pH 6.8, glycerol, 10% SDS, bromophenol blue, beta-mercaptoethanol, deionized water) for RNA and protein extraction, respectively.

### RNA preparation and quantitative PCR analysis

Total RNA with elution volume of 15 μl was obtained using the combination of 4-Bromoanisole phase separation followed by silica-based RNeasy purification (Qiagen, Hilden, Germany) according to the manufacturer’s protocol. mRNA was specifically reverse-transcribed using M-MLV reverse transcriptase system (Invitrogen, Paisley, UK) in the presence of oligo-dT primer. cDNA was diluted 5x and 0.5 μl used in qPCR. In qPCR analysis, RNA expression was quantified in triplicate reactions in a final volume of 3 μl in 384-well plates using QuantiTect SYBR Green PCR master mix (Qiagen, Hilden, Germany) following the manufacturer’s recommendations, on a LightCycler 480 (Roche Applied Science, https://www.roche.com/). qPCR reactions were prepared with the assistance of Nanodrop II liquid dispenser (Innovadyne Technologies, Rohnert Park, CA). qPCR was performed under the following conditions: denaturation (95°C for 15 min) and 45 amplification cycles (95°C for 15 s, 58°C for 30 s and 72°C for 30 s). Resulting melting curves were analyzed to test the product specificity. Each couple of primers (Table 1, Generi Biotech, Hradec Kralove, Czech Republic) at 10 pmol was used per reaction. Primers specific to 5 target genes, coding for cytokines with pro- and anti-inflammatory properties, and 3 reference genes were used for simultaneous measurements of gene expression activity. Among the candidate reference genes, TBP-1 was evaluated as the most constitutively expressed gene in our samples using RefFinder tool (http://www.leonxie.com/referencegene.php) and was selected to adjust mRNA measurements. From the obtained data, relative expression of each target gene was calculated according to the formula [1/(2^target gene Ct^)]/[1/(2^reference gene Ct^)] [36].

**Table 1:** **Gene specific primers used to assess the anti-inflammatory effect of BAF**

Gene	Primer sequence (5′ - 3′)	Gene characteristic/Primer reference
IL-1β/LAF^a^	*F:* GGGACTTGAAGAGAGAAGTGG	Pro-inflammatory/[31]
	*R:* CTTTCCCTTGATCCCTAAGGT	
TNF-α/TNFSF2^b^	*F:* CCCCCAGAAGGAAGAGTTTC	Pro-inflammatory/[32]
	*R:* CGGGCTTATCTGAGGTTTGA	
CXCL10/IP10^c^	*F:* CCCACATGTTGAGATCATTGC	Pro-inflammatory/[33]
	*R:* CATCCTTATCAGTAGTGCCG	
IL-10/B-TCGF^d^	*F:* TGAAGAGTGCCTTTAGCAAGCTC	Anti-inflammatory/[34]
	*R:* CTCATCTTCATCGTCATGTAGGC	
Arg1/Type I arginase	*F:* CCAGTCCATGGAGGTCTGTC	Anti-inflammatory/[34]
	*R:* GTGTCTTCCCCAGAGATGGA	
TBP-1	*F:* AACAGTTCAGTAGTTATGAGCCAGA	Reference gene, RNApolymerase II transcription initiation/[35]
	*R:* AGATGTTCTCAAACGCTTCG	
HMBS-2	*F:* AGGATGGGCAACTCTACCTG	Reference gene, heme biosynthesis/[35]
	*R:* GATGGTGGCCTGCATAGTCT	
HPRT-1	*F:* GAGCTACTGtAATGACCAGTCAACG	Reference gene, purine ribonucleoside salvage/[36]
	*R:* CCAGTGTCAATTATATCtTCAACAATCAA	

^a^LAF = Lymphocyte-activating factor, ^b^TNFSF2 = TNF ligand superfamily member 2, ^c^IP10 = Interferon gamma-induced protein 10, ^d^B-TCGF = B-cell derived T-cell growth factor. *F* = Forward primer, *R* = Reverse primer.

### Western blot

Intracellular activation of caspase 1 and phosphorylation of PKCϵ, enzymes involved in the signalling pathways mediating inflammatory responses, were under investigation. Cell suspensions with extraction lysis buffer were subjected to 2 - 5 min incubation in boiling water followed by short freezing. Cellular proteins of processed cell extracts were separated on a 10% SDS-polyacrylamide gel electrophoresis and transferred onto PVDF (polyvinylidene difluoride) membrane. The blot was incubated in a blocking reagent (5% low-fat dry milk suspended in wash buffer containing 2 M Tris pH7.6, NaCl, 10% Tween 20, deionized water) at room temperature for 1 hour, followed by another 1 hour incubation with specific primary polyclonal antibodies: caspase 1 (diluted 1:500; Acris Antibodies, San Diego, CA, USA), phosphorylated PKCϵ (pPKCϵ; Ser729, diluted 1:500; Santa Cruz Biotechnology, Inc., Heidelberg, Germany) and PKCϵ (diluted 1:1000; Santa Cruz Biotechnology, Inc., Heidelberg, Germany), while anti-β actin mouse monoclonal antibody (diluted 1:5,000; IgG1, clone AC-15; Abcam, Cambridge, UK) was included as a loading control. Blots were washed 3 - 4 times in wash buffer and secondary antibodies donkey anti-mouse IgG (diluted 1:10,000; Jackson Immuno Research, West Grove, PA, USA) and goat anti-rabbit IgG (diluted 1:5,000; Jackson Immuno Research, West Grove, PA, USA) applied for 1 hour at room temperature. After 3 – 4 final washing steps, proteins were visualized by using ECL Western Blotting Substrate (GE Healthcare Life Sciences, Buckinghamshire, UK).

### Statistical methods

Statistical evaluation of data obtained by serological examination and differences in the pulmonary scores was carried out by the unpaired non-parametric Mann–Whitney U test while the PCR results were statistically evaluated by the paired non-parametric Friedman with Dunn’s post test using GraphPad Software Prism 3.03 [37]. P values of less than 0.05 were considered statistically significant. Results are presented as the mean (±standard deviations) and median (min-max range) of six experimental objects in *in vivo* and *in vitro* experiment, respectively.

## Results

### *In vivo*anti-inflammatory effect

#### Effect of BAP on the clinical course of pneumonia in pigs

**Figure 1:**
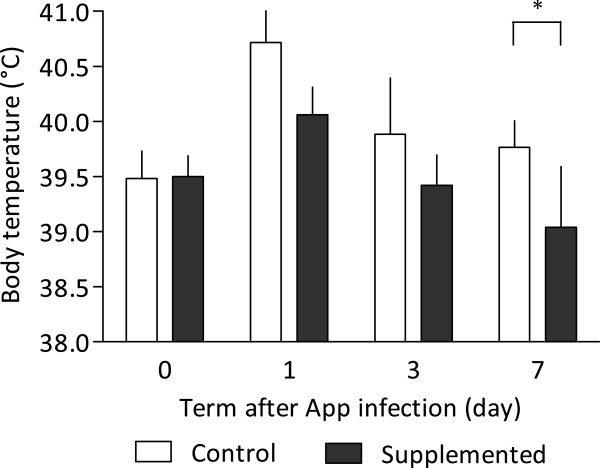
**Body temperature of pigs supplemented and non-supplemented with biologically active phospholipids after experimental infection by** ***Actinobacillus pleuropneumoniae*** **.** The data represent mean ± standard deviation of values of six animals per group. Statistical difference between groups was depicted *(*p* < 0.05).

Experimental challenge exposure to App induced infection in all pigs, but between-group differences were observed in the clinical course of infection. The animals in control group showed an increase in the respiratory rate, dyspnoea and incipient mild cough after 6–10 h PI. All 6 animals displayed increased body temperature (Figure 1), which was slowly decreasing, and remained above the physiological range in two animals after a week of infection. Comparable clinical signs were observed in supplemented group after 6–10 h PI. However, they resolved on day 2 PI (Figure 1).

### Effect of BAP on white blood cell counts in blood

As shown in Table 2, supplementation of animals with BAP before the infection did not lead to statistically significant changes in either total counts of WBC or percentages of lymphocytes and neutrophils. Increased WBC counts were detected in blood of all animals after the infection. In control group, the increase was significant (from on average 18.0 to 24.6 × 10^6^/ml, p < 0.05). Contrary to that, the increase was non-significant in the case of supplemented animals (from on average 20.1 to 21.1 × 10^6^/ml, p > 0.05). The same manner was true also for the increase and decrease of neutrophil and lymphocyte percentage, respectively. In control animals, these changes were significant (p < 0.05) in both parameters. In supplemented animals, both changes were non-significant (p > 0.05).

**Table 2:** **Effect of BAF on WBC count and differential in peripheral blood**

	WBC (x 10 ^6^in ml)		Lymphocytes (%)		Neutrophils (%)	
	***Control***	***Supplemented***	***Control***	***Supplemented***	***Control***	***Supplemented***
**Day -14**	16.5 ± 2.1	18.5 ± 4.4	66.9 ± 9.7	62.8 ± 15.8	31.2 ± 9.8	35.8 ± 9.9
**Day 0**	18.0 ± 3.2	20.1 ± 2.5	57.6 ± 7.5	58.8 ± 8.4	39.7 ± 8.1	39.4 ± 8.1
**Day 1**	24.6 ± 6.3	21.1 ± 6.5	45.2 ± 10.8	47.7 ± 11.9	52.5 ± 11.5	50.0 ± 13.1
**Day 3**	20.9 ± 6.0	21.5 ± 4.0	50.9 ± 12.9	43.1 ± 8.8	46.7 ± 13.2	55.0 ± 9.2
**Day 7**	19.2 ± 4.1	15.6 ± 2.7	54.6 ± 16.0	52.9 ± 7.8	42.8 ± 15.9	45.5 ± 8.6
**Day 14**	15.4 ± 4.4	10.6 ± 1.3	56.3 ± 18.9	64.6 ± 8.0	43.2 ± 19.1	34.4 ± 8.5

Day -14 = beginning of BAF supplementation. Day 0 – App challenge.

Values represent mean ± S.D., n = 6 animals in each group.

### Effect of BAP on white blood cell counts in bronchoalveolar lavage fluid

In BALF samples from the respiratory tract of healthy pigs, normal WBC counts are in the range of 0.8–5 × 10^6^/ml. As expected, counts of WBC in our experiment were higher in both groups than normal values. The counts achieved 11.7 ± 6.1 and 6.4 ± 4.2 × 10^6^/ml (p < 0.05) in animals from control and supplemented group, respectively.

While in healthy BALF macrophages represent 85–98% of WBC, in our experiment, these percentages dropped to 62.5 ± 13.5 and 62.7 ± 19.5 (p > 0.05) in animals from control and supplemented group, respectively. Percentage of lymphocytes increased from normal 5–10% up to 14.5 ± 6.3 and 16.6 ± 5.1 (p > 0.05) in animals from control and supplemented group, respectively. The most marked changes when compared to normal 0–5% were detected in percentage of neutrophils. Their percentages increased up to 22.7 ± 14.2 and 20.9 ± 10.6 (p > 0.05) in animals from control and supplemented group, respectively.

### Effect of BAP on serological parameters and cytokines

**Figure 2:**
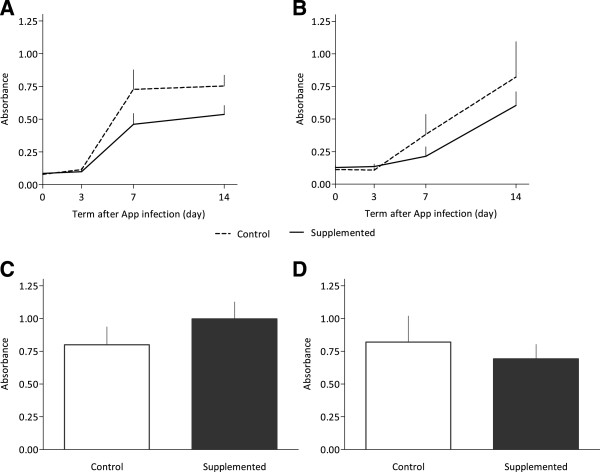
***Actinobacillus pleuropneumoniae*** **-specific antibodies in pigs supplemented and non-supplemented with biologically active phospholipids after experimental infection by** ***Actinobacillus pleuropneumoniae*** **.** Dynamics of IgM **(A)** and IgG **(B)** in serum, and levels of IgA **(C)** and IgG **(D)** in bronchoalveolar lavage fluid 14 days post-infection. The data are expressed as mean ± standard deviation of values of six animals per group.

Levels of App-specific serum IgM and IgG antibodies and local IgA and IgG antibodies in BALF were measured in the study (Figure 2). The App infection caused a typical primary immune response in the blood of pigs of both groups, a rapid onset of production of system IgM antibodies followed by production of IgG antibodies. Nonsignificantly higher levels of antibodies were observed in the control group. The examination of BALF revealed that the infection elicited IgA antibody production in the respiratory tract mucosa, being nonsignificantly higher in supplemented animals. Furthermore, a statistically nonsignificant elevation of IgG antibodies was detected in BALF in the control group.

Three animals in the control group responded by detectable production of cytokine IL-1β on day 1 PI, whereas supplemented animals did not respond to BAP at all (data not shown). No elevation of cytokine IL-8 level occurred in any of the animals in both groups tested.

### Effect of BAP on porcine pulmonary parenchyma examined post-mortem

**Figure 3:**
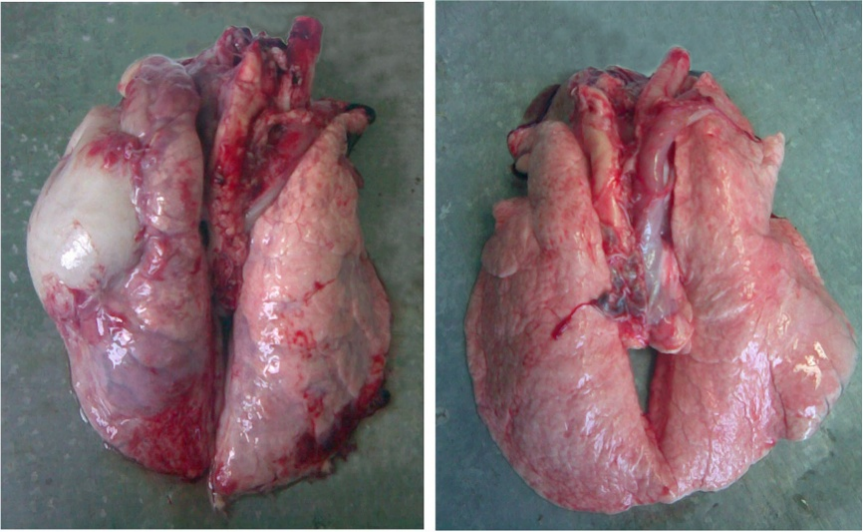
**Illustrative picture of lungs of pigs supplemented and non-supplemented with biologically active phospholipids 14 days after challenge by** ***Actinobacillus pleuropneumoniae*** **.** Lungs with pathological changes **(left picture)** were seen in pigs of non-supplemented group, whereas no significant changes were seen in lungs of pigs from supplemented group **(right picture)**.

Post-mortem examination of the pulmonary parenchyma showed a significant difference in resistance to App between the supplemented and the control group, with the percentage of inflamed tissue being 4% and 34%, respectively. The occurrence of pulmonary parenchymal lesions in the control group was chronic in nature. Necrotic-fibrinous pneumonia was diagnosed, which either was or was not associated with pleurisy and pericarditis. Figure 3 shows significant differences between the lungs of pigs in the two tested groups.

### *In vitro*anti-inflammatory effect

#### Effect of BAP on LPS-induced gene expressions in monocyte-derived macrophages

**Figure 4:**
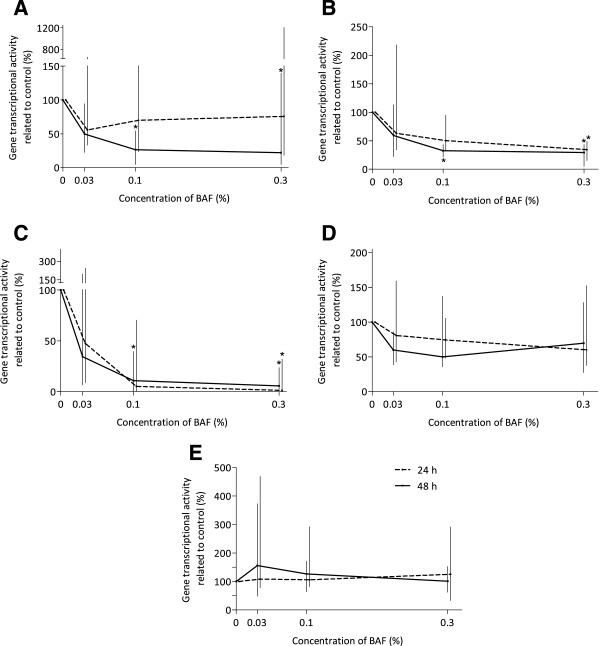
**Transcription activity of genes regulating inflammatory processes in porcine monocyte-derived macrophages after LPS-stimulation** ***in vitro*** **.** The expression activity of pro-inflammatory genes TNF-α **(A)**, IL-1β **(B)**, CXCL10 **(C)** and anti-inflammatory genes IL-10 **(D)** and Arg1 **(E)** was influenced by concentration of biologically active phospholipids and the time of action. This influence was expressed as a percentage of the control (without biologically active phospholipids) in the ratio of transcriptional activity in LPS-stimulated and nonstimulated macrophages. Values are expressed as median ± maximum and minimum of hexaplicates. Statistical difference between treatments and control was depicted *(*p* < 0.05).

To investigate the anti-inflammatory effect of BAP at the cellular level, the transcriptional activity of cytokine genes with pro-inflammatory (TNF-α, IL-1β, CXCL10) and anti-inflammatory (IL-10 and Arg1) properties induced by LPS in MDMF was evaluated. The results of this study showed that despite great individual differences, BAP is able to influence the immune response of macrophages. This influence was effectively expressed as a percentage of the decrease/increase (compared with the control) in the ratio of transcriptional activity in LPS-stimulated and nonstimulated macrophages. Their incubation with BAP led to suppression of the expression levels of pro-inflammatory cytokines and IL-10. Dose-dependent statistically significant and time-dependent expression level decrease was observed. On the contrary, Arg1 expression level was either upregulated or – in case that BAP concentrations were higher - remained the same (Figure 4).

### Effect of BAP on the LPS-induced activation of caspase 1 and pPKCϵ in monocyte-derived macrophages

**Figure 5:**
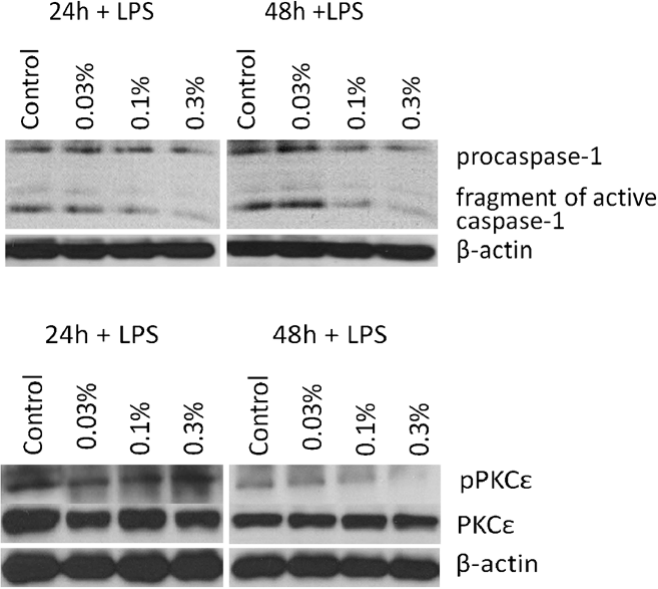
**Downregulation of caspase-1 activation and PKCϵ phosphorylation in porcine monocyte-derived macrophages by biologically active phospholipids.** The activation of caspase-1 and phosphorylation of PKCϵ decreased after 0.3% BAF incubation comparing with control (without biologically active phospholipids) and cells treated with lower concentrations (0.03% and 0.1%) of biologically active phospholipids. A higher anti-inflammatory effect of biologically active phospholipids was observed after 48 h of BAF incubation. β-actin served as loading control.

Similarly, it was found that BAP downregulated procaspase 1 fragmentation, and consequently formation of active caspase 1 after LPS treatment, depending on BAP concentration and the time of incubation. A similar effect of BAF was also detected in the case of PKCϵ phosphorylation (Figure 5). A higher anti-inflammatory effect of BAP was observed after 48 h of incubation.

## Discussion

The ability of EP to inhibit or prevent the growth and spread of tumours has been shown in many studies [3, 6, 9, 23, 38]. Even so, the exact mechanism of action leading to suppression of neoplastic cells has not been completely clarified. Recently, it was proved that EP in plasma membrane act on ion channels, modulate their function [39], and consequently enhance calcium ion cell concentration altering cell membrane permeability finally leading to death of tumour cell [23]. Contrary to this finding, the inflammatory response of the host immune system to bacterial infection or tissue injury can trigger or accelerate a neoplastic process [40]. Therefore, safe compounds with the anti-inflammatory potential are needed in therapeutic treatment and prophylaxis of inflammation-related diseases including atherosclerosis, inflammatory arthritis and even neoplasm. The reducing effect of natural compounds on inflammation by suppression of inflammatory cytokines *in vivo* and *in vitro* applicable in such cases has been described [40–43]. However, the effect of EP on the immune system response has not been well studied yet. The immunostimulatory and regenerative effects of derivatives of EP on the immune system were demonstrated in studies, in which mice [44, 45] and pigs [46] served as experimental models. To the best of our knowledge, no previous studies have reported the transcription cell response to EP.

In the present study, porcine system was used for the first time to evaluate the anti-inflammatory and immunomodulatory response to BAP after App- or LPS-activation of the immune system. *In vivo,* a milder course of the disease and easy recovery of animals showed a potent protective effect of BAP and indicated the importance of BAP in inflammation development. Furthermore, the parameters of the innate and adaptive immune defence against the infection including neutrophils and macrophages as non-specific immune cells, and specific immunity mediating antibodies and lymphocytes, confirmed the apparent protective potency of the compound. Specifically, changes in total and differential counts of WBC and bronchoalveolar cytology reflecting ongoing bacterial infection confirmed the assumed immunomodulatory and anti-inflammatory effect of BAP. BAP-supplementation led to a slight increase in WBC count in the blood. Furthermore, the investigated parameters more quickly returned to normal values due to BAP.

A similar boosting effect on WBC count, but statistically significant, was demonstrated by bronchoalveolar lavage cytology, with considerable differences in lymphocyte and macrophage percentages between the tested groups. This is in accordance with other authors who observed an increased non-specific immune response [46] in a bacterial stimulus free diet experiment, in which choline based EP, soy or egg lecithin were orally administered to weaned piglets. An increase of granulocyte and decrease of lymphocyte percentages in blood observed on day 11 of supplementation of each of the lecithins were comparable to those obtained after 14 days of BAP-supplementation immediately before infection. Moreover, the maximum immunomodulatory effects of choline- and BAP-based preparations on WBC differential recognised on the identical day 18 of supplementation in each preparation tested were found to be comparable, irrespective of the ongoing infection in the BAP experiment. Furthermore, our serological results showed that only pigs non-supplemented with BAP responded by detectable IL-1β and IL-8 production and thus confirmed the ability of BAP to inhibit the inflammatory process. In supplemented pigs, BAP also contributed, statistically significantly, to a lower damage to the pulmonary parenchyma without further complications than was observed in unaffected pigs.

To verify anti-inflammatory potential of BAP in macrophage system, we further analysed gene expression profiles of cytokines and elevation of other mediators involved in the inflammatory process. Generally, the cytokine profiles were in agreement with our previous findings. Macrophages clearly responded to the tested compound by downregulation of expression of proinflammatory cytokines. However, the effect of BAP on expression of anti-inflammatory cytokines was not uniform. According to established downregulation of IL-10 expression we can deduce that BAP tends to lead to homeostatic balance of proinflammatory and anti-inflammatory cytokines, which is crucial for maintaining health. On the other hand, expression of arginase, which was shown to participate in inflammation-triggered immune dysfunctions, immunosuppression and immunopathology of infectious diseases, was influenced rather positively by BAP treatment. Specifically, arginase expression in macrophages is regulated by different stimuli such as LPS, lipoproteins, inflammatory stimuli and hydrogen peroxide in a species-specific way, and the enzyme function in the immune system of mammals except pigs has frequently been studied [47]. Similarly to EP capable to inhibit PKC [15, 16], we demonstrated the inhibition of phosphorylation of PKCϵ by BAP. Also, downregulation of procaspase 1 fragmentation demonstrated here is in accordance with the aforementioned results confirming the anti-inflammatory potency of BAP.

## Conclusion

In this study, under *in vivo* conditions*,* we detected anti-inflammatory and immunomodulatory effects of BAP at all levels investigated: health status, total and differential counts of WBC, serological parameters, pro-inflammatory cytokines, BALF and pulmonary parenchyma. Furthermore, BAP exerted anti-inflammatory activity *in vitro* under controlled conditions of monocyte-derived macrophages stimulated with LPS. It manifested itself as modulation of gene expression of cytokines and activation enzymes playing an important role in the activation of intracellular signalling pathways associated with the induction of inflammation.
